# Design factors that influence PCR amplification success of cross-species primers among 1147 mammalian primer pairs

**DOI:** 10.1186/1471-2164-7-253

**Published:** 2006-10-09

**Authors:** Donna JE Housley, Zachary A Zalewski, Stephanie E Beckett, Patrick J Venta

**Affiliations:** 1 Small Animal Clinical Sciences, Michigan State University, East Lansing, MI 48824-1314, USA; 2 Microbiology & Molecular Genetics, Michigan State University, East Lansing, MI 48824-4320, USA

## Abstract

**Background:**

Cross-species primers have been used with moderate success to address a variety of questions concerning genome structure, evolution, and gene function. However, the factors affecting their success have never been adequately addressed, particularly with respect to producing a consistent method to achieve high throughput. Using 1,147 mammalian cross-species primer pairs (1089 not previously reported), we tested several factors to determine their influence on the probability that a given target will amplify in a given species under a single amplification condition. These factors included: number of mismatches between the two species (the index species) used to identify conserved regions to which the primers were designed, GC-content of the gene and amplified region, CpG dinucleotides in the primer region, degree of encoded protein conservation, length of the primers, and the degree of evolutionary distance between the target species and the two index species.

**Results:**

The amplification success rate for the cross-species primers was significantly influenced by the number of mismatches between the two index species (6–8% decrease per mismatch in a primer pair), the GC-content within the amplified region (for the dog, GC ≥ 50%, 56.9% amplified; GC<50%, 74.2% amplified), the degree of protein conservation (R^2 ^= 0.14) and the relatedness of the target species to the index species. For the dog, 598 products of 930 primer pairs (64.3%) (excluding primers in which dog was an index species) were sequenced and shown to be the expected product, with an additional three percent producing the incorrect sequence. When hamster DNA was used with the single amplification condition in a microtiter plate-based format, 510 of 1087 primer pairs (46.9%) produced amplified products. The primer pairs are spaced at an average distance of 2.3 Mb in the human genome and may be used to produce up to several hundred thousand bp of species-specific sequence.

**Conclusion:**

The most important factors influencing the proportion of successful amplifications are the number of index species mismatches, GC-richness of the target amplimer, and the relatedness of the target species to the index species, at least under the single PCR condition used. The 1147 cross-species primer pairs can be used in a high throughput manner to generate data for studies on the genetics and genomics of non-sequenced mammalian genomes.

## Background

Cross-species polymerase chain reaction (PCR) primers are often used to amplify part of a gene or genome for which no direct species-specific sequence information is available. There are a number of uses for these primers including the development of genetic maps for linkage analysis, identification of genetic markers for use in conservation biology, development of RH maps in studies of chromosomal evolution, and sequence comparisons for studies of molecular evolution for species whose genome sequences have not yet been determined [1-5]. The simplest method of producing cross-species primers is to design primers to a region of a genome for a given species and then to empirically test these for amplification in other species. For example, primer pairs designed to flank avian microsatellite repeats in one species have been used to amplify homologous sequences in other closely related avian species [6]. A limitation of this method is that it is not possible to predict the likelihood that any given primer set will work in another species because the evolutionary history of the primer binding region across species is unknown. However, such primer sets have been shown to be useful for the amplification of the homologous target in relatively closely related species [7]. Not surprisingly, there is a strong correlation between the proportion of sets that work in other species and the genetic distance of those species from the species for which the primers where designed in the first place. It has been estimated that 50% of these primer sets will work for avian species that have diverged by 11 MY (millions of years) for passerines and 23 MY for non-passerines [7].

It is known, in a general sense, that the more mismatches there are between a primer and a target, the less likely the target is to PCR-amplify. A way to reduce the probability of mismatches between primers and targets is to pick regions of the genome that are more conserved than those picked more or less at random. Coding regions of genes are known to be more conserved than intronic or intergenic regions. An early variation of this approach was to use a computer program to design primers in coding regions of genes judged to be optimal for amplification in one species, and then test these primers in other species [8]. Although the program would often pick identical or nearly identical regions between primates and rodents, the choice of regions conserved between species did not appear to be a primary design consideration. Another approach is to design degenerate primers to conserved coding regions. However, it is generally acknowledged that degenerate primers often result in amplification of not only the desired target but also of artifactual bands and non-orthologous homologues (indeed, amplification of multiple family members is often the point of this powerful technique; [9]). However, if the goal is to amplify a single target gene in a family, say for the purpose of building a genetic map, great care is needed to identify the desired product among multiple bands in contrast to unique-sequence primers that often produce only a single band.

Use of conservation of sequence as a design consideration for unique sequence cross-species primers has been used by many laboratories for small numbers of cross-species primers used for specific targets (e.g., [10,11]). However, at least three larger panels of cross-species primers have been developed for more general use in mammals [12-14]. The primer pairs in these panels often require some degree of reaction optimization that has hindered their wider use. In order to increase the efficiency with which cross-species primer panels can be used, we have developed a new panel of mammalian cross-species primers which can be used to examine the influence of various design parameters, and for which a reasonably predictable proportion will work for any given mammalian species under a single PCR condition. Although it is thought that inexpensive whole genome sequencing, perhaps as low as $1,000 per genome, will be available in a few years, cross-species primers may still be useful for studies in which only a sample of a genome is needed and for which off-the-shelf reagents are found to be convenient to obtain the sample [2]. The results concerning the most influential factors in cross-species primer design should also be of use in designing primers pairs for studies on other major branches in the tree of life.

## Results

### Summary information on primer pairs

**Figure 1:**
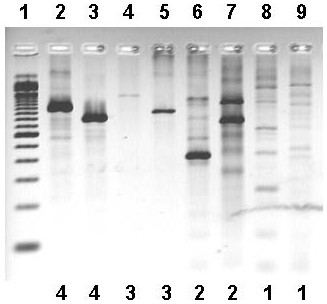
Examples of PCR results ascribed to subjective rankings used for cross-species amplifications shown in Table 3. Lane numbers and subjective rankings appear above and below the picture, respectively. Lane 1, 100 bp ladder (Invitrogen Corp.); lanes 2 and 3, "4" ranking (lane 2, *MACF1 *monkey; lane 3, *MACF1 *dog); lanes 4 and 5, "3" ranking (lane 4, *GNB1 *mouse; lane 5, *ORC4L *cat); lanes 6 and 7, "2" ranking (lane 6, *DVL1 *goat; lane 7, *CDC7 *hamster); lanes 8 and 9, "1" ranking (lane 8, *DVL1 *monkey; lane 9, *DVL1 *pig). Ranking numbers are also provided below each lane. A ranking of "0" signifies that no bands of any kind were seen (not shown).

The summary statistics of the cross-species primers are reported in Table 1. The total intronic region that would be theoretically PCR-amplified in the human genome by these primer pairs is 1,100,272 bp, and the coding region covered is 143,282 bp (excluding the primer binding regions). Seventeen pairs (noted in Table S1 [Additional file 1]) have not been tested in any species, but were designed in the same way as all of the other primer pairs and provide additional coverage for some regions of the genome. Products were inferred to be the intended target if they were at least 70% identical to the intended target and had splice signals in the same position as the human gene. Several hundred of these products were as BLASTed against the nonredundant Genbank database (prior to the release of the canine genome sequence) and in all cases the most significant sequence match was either an index species sequence used to design the primers or an ortholog of that sequence from another species. Of the 1147 primer pairs reported (58 previously reported [4]), 1016 were tested on dog genomic DNA and 637 (62.7%) produced products that were shown to be the intended target by sequence analysis (Table S1 [Additional file 1]). If the 81 primer pairs for which dog was an index species are excluded, the success rate was 64.0% (598/935). An additional 3% produced sequences that did not match the intended target. For the hamster, 1087 primer pairs were tested under a single reaction condition in microtiter plates. The hamster products were not sequenced, but based upon subject rankings derived from the experience with the canine products, 510 (46.9%) resulted in amplifications with a subjective ranking of 2, 3, or 4 that were judged to be likely to contain the correct product (see Fig. 1 for an explanation of the ranking system and Table S1 [Additional file 1] for individual rankings). For a ranking of 2 in which an extra band is seen, more than 95% of the correct canine bands are closer to the human size than the artifactual band (data not shown). Less than 11% of the hamster amplifications deemed to be successful amplifications have a ranking of 2 (Table S1 [Additional file 1]).

### Empirical determination of the impact of index-species mismatches on the percentage of successful amplifications

Two types of nucleotide mismatches are of interest in this work. The first type of mismatch is that found between the sequences of the two index species in the target region to which a primer is designed (as noted in methods and materials, the primer itself always exactly matches one of the index species). These mismatches are designated as index-species (IS) mismatches. When an IS-mismatch is allowed, the primer is designed under the assumption that the sequence of one the index species (the primary index species; usually human) represents the nucleotide in the common ancestor. The second type of mismatch is the primer-template (PT) mismatch, in which a nucleotide difference is known to exist between a given primer and a given (sequenced) target DNA.

At the beginning of this work, some primer pairs were designed that allowed one or more IS-mismatches in order to save labor during the primer design step. It was known from previous results that IS-mismatched primers often worked [12]. However, after 245 primer pairs had been developed (for genes found on human chromosomes [HSA] 1, 2, 11, 17, 19, and 22), the data was analyzed to determine the impact of IS-mismatches on the percent of primer pairs that would amplify the correct canine gene (the canine genome sequence was not available at the beginning of this work, and so PT-mismatches for the dog could not be identified at that time). For this sample it was found that 60% (66/110) of the primer pairs amplified dog DNA if no IS-mismatches were present in either of the forward and reverse primers, but that only 47% (61/131) amplified if at least one IS-mismatch was present. This result suggested that IS-mismatches have an important impact on amplification success, so greater effort was made to make as many primer pairs exactly match the two index species as possible. Analysis of the 1016 primers designed without the dog as an index species indicated that there was a significant difference (p = 0.00055) between the amplification efficiency of primer pairs with no IS-mismatches (548/841, 65.2%) vs. primers pairs with one or more IS-mismatches (90/175, 51.4%) (Table 2). For the 1087 primer pairs tested with hamster DNA, amplified products (2, 3, or 4 ranking in which it was subjectively judged that the correct product amplified; see Fig. 1) were obtained for 443/905 (49.0%) of those primers with no IS-mismatches, but only 67/182 (36.8%) of those with one or more mismatches (p = 0.004; Table 2). Regression analysis of percent of targets successfully amplified vs. number of IS-mismatches suggested a decrease of 6–8% in the success rate with each additional IS-mismatch.

Subdividing the IS-mismatch primers into sets with different numbers of IS-mismatches (1, 2, or 3) or by distribution of mismatches (e.g., one IS-mismatch in each of the forward and reverse primers of a set, vs. two IS-mismatches in the forward, but none in the reverse) did not show a statistically significant difference in success rate. The lack of a difference could be because the sample sizes were small for primer pairs with one or more IS-mismatch.

### Effect of primer-template mismatches on amplification success

The release of the assembled canine whole genome sequence provided an opportunity to assess the impact of the number of PT-mismatches on the success of target amplification. Twenty primer pairs that amplified the correct dog target and 20 primer pairs that produced no product under the standard conditions were aligned with the homologous canine sequence (Tables S2 and S3 [Additional file 2]). For the 20 that did amplify, all but one had two or fewer PT-mismatches. For the 20 that did not produce a dog product, all but seven had 3 or more total PT-mismatches between the primers and the dog target. When the sequences for the three genes that had only zero or one mismatch but did not amplify were examined more carefully, it was noted that they contained all or part of a GC-rich island. All 40 genes were tested again in the presence of 1 M betaine (a known enhancer for amplification of GC-rich sequences), and the three high GC-content genes with zero or one mismatches produced a product, although none of the primer pairs with three or more PT-mismatches produced a band. These results with the canine PT-mismatches suggest that a relatively sharp cutoff in amplification success occurs around two to three total PT-mismatches per primer pair under the standard PCR conditions and for primers averaging 22 bases in length (Table 1).

### The effect of the other factors examined

In order to determine the impact of the other factors examined on the rate of amplification success, a sample of 50 primer sets were examined across five mammalian Orders (primates; humans and macaques: carnivores; dogs and cats: perissodactyls; horse: artiodactyls; cows, goats, and pigs: and rodents: hamsters, rats, and mice (Table 3) and, in certain instances as described below, across the complete set of primers as tested using only dog and hamster DNA.

Among the sample of 50 primer sets tested with the two index species' DNA (100 tests), it was unexpectedly observed in 14 tests that the DNA from the index species (known to have no PT-mismatches) failed to amplify or amplified poorly (i.e., had a ranking less than 3; Table 3). In nearly all cases, it appears that this was either due to the presence of a processed pseudogene (as inferred by the presence of a band that closely matched the size predicted for the cDNA [given in Table S1 {Additional file 1} for each target]) that served as significant competition for amplification of the intended target, or because the region amplified was GC-rich or contained a patch of at least 130 bp that was GC-rich (>59% G + C - often part of a GC-rich island). A correspondingly lower overall success rate was generally observed across the full set of species for which one of the index species was found to have a GC-rich template (e.g., *BIN1 *and *DVL*, found in the lower half of Table 3).

When the full set of 841 zero IS-mismatch primer pairs was analyzed for the dog, 74.2% of targets with a human GC content of <50% amplified, whereas only 56.9% of targets with a GC content of ≥ 50% amplified (p = 1.9 × 10^-7^) (Table 4). However, when the same analysis was done for the hamster, no significant difference was detected between the two groups of targets (p = 0.64). In order to provide evidence that this might be due to GC content differences between the homologous targets of the dog and hamster, the GC content was determined for the 30 targets with zero IS-mismatches between human and mouse (a rodent for which sequence is available) for which the human target had a GC content ≥60%. The result was that the mouse target had, on average, 6.9% less G+Cs than the human target.

Because of the impact of human GC-content on the amplification efficiency for some primer pairs for some species, we made an empirical assessment of the impact of including 1 M betaine on the percent of successful amplifications compared to the standard conditions using a non-rodent, non-primate species. Primer pairs for 87 genes on HSA6 were used to amplify pig DNA in a microtiter-plate format. Under standard conditions, 39 pairs produced a band of the expected size (34 as single bands that should be amenable to direct sequence analysis). Bands appeared for twelve additional genes when 1 M betaine was included (all single bands in this experiment). However, the bands for 6 other pairs (all single bands) that were present under the standard conditions disappeared with the inclusion of betaine (data not shown).

Several other parameters were examined for a possible effect upon amplification efficiency by examining the results obtained with the sample of 50 primer pairs among the species from five mammalian Orders. The parameters included primer length, sequence identity between the human and mouse proteins encoded by each gene, and CpG-content of the primer region. No significant effect of primer length on amplification success was observed. A modest, but statistically significant, effect (R^2 ^= 0.14, p = 0.016) was observed for sequence identity on proportion of species amplified. The effect of CpGs on amplification success was examined because it is known that CpGs are, in general, hotspots for mutation depending upon their methylation status and might therefore be expected to cause a greater number of PT-mismatches for primers that overlie CpGs [15]. However, no statistically significant effect on amplification success was found for CpG content within the sample of 50 primer pairs used with the DNA from the 11 species. The effect of CpGs in the primer region was also examined in the complete data sets for the dog and hamster DNA and again no statistically significant association was found.

It was also observed that there tended to be a higher success rate for species within the same Order as one of the index species as compared to species in Orders other than those of the index species, as would be expected and as previously shown for randomly designed primers [6]. For example, for the 24 primer pairs for which the mouse was an index species, 24 of the rat and 23 of the hamster targets amplified, but only 14 of the cow, 14 of the goat, and 16 of the pig targets amplified (p ≤ 0.01 for within- vs. between-Order amplification).

## Discussion

The goals of this work were to determine what factors influence the success rate for cross-species primers and to develop a set of cross-species primers that would cover most of any mammalian genome and that could be used under a single standard reaction condition with a predictable rate of success. Of the 1147 primer pairs designed, 955 (83.3%) have zero IS-mismatches. Of those tested in the dog, 65.2% of the targets were amplified and verified to be the correct target by sequence analysis (Table 2). These were amplified under a single amplification condition, suggesting that the primer pairs should be useful for high throughput data collection for un-sequenced mammalian genomes. Although 16.7% of the 1147 primer pairs have one or more IS-mismatches, they still produce correctly amplified products in about 36.5% of hamster targets and 49% of the canine targets. The single condition that we used was chosen because it has generally worked well in our lab for cross-species primer work.

Before examining the importance of the design factors studied, it may be helpful to briefly discuss the rationale we used to design these primer pairs. When the sequences for two index species are aligned, we assume that identical nucleotides have not changed from the common ancestral sequence. Once an ancestral sequence has been inferred, the rate of evolution from the ancestor to the target species (i.e., the number of substitutions that are fixed in the primer binding regions) is then a major determining factor of amplification success. As a corollary, if a stretch of sequence is identical, it is only necessary to align two index species sequences to infer the sequence of the common ancestor. Incorrect inference occurs in the case of parallel substitutions (or other types of molecular evolution that lead to homoplasy) when using sequences of only two index species [16]. The sequences of five divergent species are required to be able to recognize most cases of parallel substitution and, because parallel substitutions are relatively rare, the gain from the additional work to obtain multi-species alignments would only marginally increase the success rate of cross-species primers.

With regard to the other factors examined, no statistically significant correlation was found between amplification success and the length of primers (between 18 and 30 bases), and with the number of CpG dinucleotides contained in the primers. A statistically significant effect was found with the degree of sequence identity between the proteins of human and mouse. However, the predictive value (R^2 ^= 0.14) is relatively modest, and it may not be worth the effort to use this factor for the selection of gene targets.

The impact of GC-richness in a target is a significant determinant of amplification efficiency for the dog, and probably other species with the common mammalian GC content pattern, at least under the standard conditions used in this work. However, it may be difficult to avoid using these targets if samples for all segments of a mammalian genome are desired as, for example, when developing markers for whole genome linkage scans. This is because large stretches (isochores) of most mammalian genomes tend to be GC-rich and it may not be possible to find a conserved region that does not reflect this GC-rich condition. There also tends to be a correlation between high gene density and GC-richness in mammalian genomes, and it will generally be necessary to identify polymorphisms in these regions [17,18]. Use of a reaction condition that increases the amplification efficiency of GC-rich targets, such as the inclusion of 1 M betaine, can be used for regions where this problem is encountered. By using this one additional reaction condition, an estimated 5–10% of additional targets will amplify; however, a roughly equivalent number of bands compared to the standard condition may be lost, so it is best not to use the betaine condition by itself. Other reaction conditions (e.g., different cycling conditions, reactant concentrations) were not examined systematically or in any detail in this work.

In contrast to the canine genome, there is a lack of an association for the hamster between the human GC content of a target and the amplification success rate. One explanation for this striking contrast may be that the hamster genome, like the mouse genome, has significantly less GC-rich regions (i.e., those over 50% G+C) than the human and most other non-rodent genomes. This "murid shift" in GC content appears to be specific to rodents in contrast to the general mammalian pattern [19,20]. We verified that the mouse targets for the most GC-rich human genes are much less GC-rich by an average of 6.9% (e.g., a human target that is 60% GC would only be 53% GC in the mouse). At least one report in which two particular genes of rodents were studied provides data suggesting that the GC-content of hamster DNA may be 3% lower than that of the mouse, and this is in keeping with the lack of a significant effect of human GC content as a predictor of hamster PCR success rate [21]. Further work will be needed to directly verify the lower GC content in the hamster targets. We cannot explain the overall lower success rate of the hamster targets as compared to the canine targets, although perhaps our microtiter plate format is somewhat less efficient than our single tube format. Further work will also be needed to explain this difference.

As one would certainly expect, PT-mismatches and IS-mismatches (as predictors of PT-mismatches) are important determinants of the probability that a target will amplify. The data reported here provide: (1) an estimate of the number of PT-mismatches that will still allow amplification to occur (generally no more than 2 under the conditions reported) and (2) an estimate of the impact of IS-mismatches on the probability of amplification success (e.g., a decrease in success rate for hamster DNA from 49.0% for no IS-mismatches to 19.2% for three IS-mismatches). Under the standard conditions used here, we observed that if two or fewer primer-binding region substitutions occur during the evolutionary period from the common ancestor to the target species, amplification is likely, and if three or more substitutions occur, amplification is unlikely. We have observed cases where three or more PT-mismatches will still allow amplification, but these are relatively rare (e.g., *NUP53 *in Table S2 [Additional file 2], and Housley and Venta, unpublished observations). The data presented here were not sufficient to determine of what importance the distribution of PT-mismatches within the forward and reverse primers might have on amplification efficiency.

Based upon the simplifying assumptions that mutations occur randomly, that non-synonymous changes do not occur in conserved regions, and that the fixation of synonymous mutations follows a Poisson distribution, the proportion of primer binding regions that will have three or more mismatches can be calculated based upon the rate of evolution for the gene in question between the two index species. Despite the obvious oversimplification, a preliminary analysis suggests an approximate fit between the predicted and observed data. A broader implication for this simple model is that it should be possible to select index species based upon the rate of divergence in coding regions for any group of organisms (e.g., plants, animals, microorganisms) that will provide a predictable rate of success prior to designing primers. Degree of divergence between representative genomes of a clade (say perhaps chicken and zebra finch for birds) might therefore be included among the criteria for selecting which genomes to sequence next.

Although it is probably possible to obtain a marginal increase in the proportion of amplified products that can be obtained by using multiple reaction conditions, we suggest that it may be more efficient to use a single condition, such as the one reported here, to maximize the amount of data obtained for a given amount of effort and time. If a somewhat higher success rate is desired, we suggest that one additional condition be used, that being the inclusion of an enhancer such as betaine for GC-rich targets. If the whole set of 1147 primer pairs is not needed, then the primer pairs contained in Table S1 [Additional file 1] can be sorted to select a desired combination of properties (e.g., only pairs with no IS-mismatches, GC-content below 50%, greatest amount of coding region obtained, etc.).

There are several areas of research in which cross-species primers have been of value. For example, they have been used for the construction of physical maps of genomes by radiation hybrid (RH) mapping and/or genetic maps by identifying genetic variation in the amplified products [4,22,23]. Construction of these maps by using these primers might be particularly useful for studies of chromosomal evolution [24]. The amplification information provided here on the hamster targets may help in the selection of primer pairs for radiation hybrid mapping because hamster cells are most often used as the recipient for RH work [4,22,25]. Some researchers prefer primer pairs that do not amplify the potentially interfering hamster band (and may even develop new species-specific primers after determining the sequence of the amplified product), while others prefer to have presence of the hamster band as an internal amplification control [26,27]. Cross-species primers have also been used successfully for other purposes, such as the study of the molecular evolution of organisms [11,28,29]. Indeed, it has been suggested that the sequences of nuclear genes may provide more accurate information for studies of evolution and conservation compared to the more heavily used sequences of mitochondrial DNA [30].

The choice between whole genome sequencing, EST sequencing, or use of cross-species primers will depend upon the goals and resources available for each research project. Cross-species primers will remain cost-effective for projects involving comparison of multiple species because a single set of primers can be utilized to amplify across many related genomes, without the need to design and synthesize individual primer pairs for each different species. However, once the methods for producing the sequence of a complex genome for $1,000 have been realized, the value of cross-species primers will be greatly diminished in instances where a single species is of interest, because it will be possible to obtain complete genome sequence information at a low cost [31]. Until this has been accomplished, however, cross-species primers may be of use for obtaining data that will lead to insights in evolution, genomics, and possibly other areas of biology.

## Conclusion

The relatively recent completion of the canine genome sequence allowed us to identify several factors that have a significant impact on the efficiency of cross-species primers for use in a high throughput fashion. These include the number of index-species mismatches, the GC-content of the target amplimer, and the degree to which a target species is related to one of the index species. On the other hand, the presence of CpG dinucleotides (with their relatively high genome-wide mutation rates), the length of the primers within the range studied (18 – 30mers), and the degree of encoded protein sequence conservation appear to have a minimal impact on the probability that a given primer set will amplify a given target. These observations should aid in developing automated systems for cross-species primer design for clades of life (e.g., non-mammalian vertebrates species such as birds, insect species such as mosquitoes, and plant species such as Orders of dicots) in which only a few model organism genomes have been sequenced. In addition, the 1147 primer sets used in the study may have immediate uses in genetics and genomics of mammalian species in which whole genome sequences are not yet available.

## Methods

### Selection of gene targets and DNA samples

Genes were selected to give reasonably even coverage across the entire human genome by use of the UCSC Genome Browser [32]. With a few exceptions, targets were chosen that had an intron size of a few hundred to 2000 bp. An attempt was made to avoid genes that tend to produce multiple pseudogenes (e.g., genes encoding ribosomal proteins; [33]). Genomic DNA samples isolated from blood, tissue, or cell culture were obtained for 11 species (human, pigtail macaque, dog, cat, horse, goat, cow, pig, Chinese hamster, rat, and mouse) representing five mammalian Orders. Other researchers provided most of these; however, the hamster DNA was isolated by a standard phenol-chloroform extraction method from a CHO cell line.

### Design of primers

Primers were manually designed to conserved regions between two species (the index species) of different mammalian orders (generally human and either rat or mouse). The 3' end of the forward primer was generally designed to overlie a second codon position and the 3' end of the reverse primer was generally designed to overlie the first codon position. Except in 6 cases, the forward and reverse primer had annealing temperatures within 4°C of one another. Eighty-four percent of the primers pairs were designed to completely conserved regions.

Occasionally IS-mismatches (defined in the Results section) were allowed in the primer-binding region, generally when no completely conserved region could be found. In most of these cases, the primer was designed to exactly match the human gene sequence because it is thought that primate molecular evolution is significantly slower than rodent evolution and the human should, therefore, be more likely to match the sequence of the common ancestor to primates and rodents [34]. Occasionally, dog was used as the second index species and primers were designed to exactly match the dog, because this species is of major interest in our lab. Predicted product sizes for the human targets were determined by using the *in-silico *PCR (ePCR) function of the UCSC Genome Browser [32]. The primer sequences, predicted product sizes from the human genome, melting temperatures, presence of amplified products from the hamster genome, and other pertinent data are contained in Table S1 [Additional file 1].

### PCR conditions

Targets were amplified from 10 to 50 ng of genomic DNA in a 25 μl volume containing 10 mM Tris (pH 8.3 at 20°C), 50 mM KCl, 100 μM dNTPs, 1.5 mM MgCl_2_, 0.08 μM of each primer, and 0.5 U Taq DNA polymerase (Invitrogen Corp., Carlsbad, CA). The PCRs for the dog targets were cycled in a MJR-100 Thermocycler for 1 min at 94°C, 2 min at 59°C, and 3 min at 72°C for 35 cycles. Fifty primer sets were tested for amplification across species using the identical single condition given for the dog amplifications. The hamster and pig DNA samples were also amplified under the same conditions, but in a microtiter plate format using a Robocycler (Stratagene, Corp., La Jolla, CA). In a limited number of cases where GC-rich targets were known or expected, 1 M betaine was added to the standard reaction [35]. These cases are specifically noted in the Results.

### Gel electrophoresis and sequence analysis

Dog amplification products were run on 2% agarose gels with standard gel electrophoresis units and stained with ethidium bromide to visualize bands. Hamster products underwent electrophoresis using a rapid agarose gel electrophoresis (RAGE) unit (Cascade Biologics, Inc., Portland, OR). PCR products were generally gel-purified using the Qiaex II gel extraction kit (Qiagen Corp., Valencia, CA), but some automated sequencing was performed directly from the amplification reactions. Manual sequencing was performed using the Thermo Sequenase Radiolabeled Terminator Cycle Sequencing Kit (USB Corp., Cleveland, OH). The identity of the sequence was confirmed by BLAST analysis [36] or, later, by BLAT analysis [32] when this program became available [37, 38]. Canine sequences were inferred to be homologous to the intended target if the most significant match identified using BLAST or BLAT was the correct gene or mRNA in any mammalian species and if the splice point(s) were in the expected position relative to the coding region.

## Authors' contributions

DJH did all of the dog amplifications, sequencing, designed primers, analyzed data, and prepared the manuscript. ZAZ performed nearly all of the hamster experiments, the pig array/betaine experiments, and analyzed this data. SEB performed the cross-species experiments contained in Table 3 and analyzed this data. PJV designed primers, analyzed the data, and prepared the manuscript.

## Figures and Tables

**Table 1:** Primer and target statistics

Parameter	value^a^
number of primer pairs	1147
number with no IS-mismatches	955
spacing in human genome	2.3 +/- 2.4 Mb (0.0 – 23.2)
product size^b^	1130 +/- 514 bp (197 – 3862)
primer length	21.8 +/- 3.1 b (16 – 38)
annealing temperature	61.0 +/- 1.4°C (55.0 – 69.0)
introns crossed	1.08 +/- 0.36 (0 – 4)

^a^For values that vary, data are given as average +/- standard deviation or range.

^b^These values are based upon the human target.

**Table 2:** Number of IS-mismatches vs. target amplification

	Dog			Hamster		
		
IS-Mismatches	Tested^a^	Product^b^	%	Tested	Product^b^	%
0	841	548	65.2	905	443	49.0
1	100	55	55.0	104	44	42.3
2	43	19	44.2	44	16	36.4
3	25	13	62.0	26	5	19.2
4 + 5	7	3	42.9	8	2	25.0

^a^These numbers do not include primer pairs for which the dog is an index species.

^b^Dog products were all confirmed by sequence analysis. Hamster products were inferred to be correct based upon a subjective ranking of 2, 3, and 4 (see Fig. 1 for ranking system).

**Table 3:** Cross-species amplification results for 50 primer pairs with no IS-mismatches

Gene	Hum^a^	Mon	Dog	Cat	Hor	Cow	Goa	Pig	Ham	Rat	Mou	prot id^b^	GC^c^	Notes^d^
PTBP2	***4***^e^	4	4	4	4	4	4	4	4	4	***4***	99	32.8^c^	
HAT1	***4***	4	4	4	4	4	4	4	4	4	***4***	93	39.6	
PTPRU	***4***	4	4	4	4	4	4	4	4	4	***4***	95	54.8	
MACF1	***4***	4	4	4	4	4	4	4	4	***4***	4	78	37.8	
FRAP1	***4***	4	4	4	4	4	4	4	4	***4***	4	99	39.9	
LRP1B	***4***	4	3	4	3	4	4	4	4	4	***4***	91	39.5	
LYST	***4***	4	4	3	3	***4***	4	4	4	4	0	84	28.6	
HNRPU	***4***	4	4	2	4	4	4	4	4	***4***	4	94	39.6	
SLC20A1	***4***	4	4	2	4	4	4	4	4	4	***4***	90	39.4	
RND3	4	4	0	4	4	4	4	***4***	4	4	4	100	51.6	
CDC7	***4***	2	4	4	4	4	4	4	2	***4***	0	81	33.6	
PABPC4	***4***	4	4	4	4	4	4	4	3	2	***2***	95	51.2	
NR4A2	***4***	4	4	3	0	4	4	4	4	***4***	4	99	43.9	
KYNU	***3***	4	4	3	4	4	4	0	4	***4***	4	85	30.5	
DDX18	***4***	4	0	3	4	4	4	4	4	4	***4***	85	39.4	
CLCN6	***4***	4	4	4	4	0	4	3	3	4	4	96	52.7	1^d^
PSCDBP	***4***	4	3	4	4	0	4	3	4	4	***4***	81	37.4	
ABCA4	***4***	4	2	4	4	***4***	4	2	3	2	3	88	53.5	
MDH1	***4***	4	4	4	4	4	2	***2***	4	2	4	96	39.5	
ADD2	***4***	4	0	4	4	4	2	4	2	4	***4***	94	51.9	
CTNNA2	***4***	4	4	4	4	0	0	4	3	4	***4***	98	45.6	
SLC1A4	***4***	4	4	4	2	2	4	2	2	3	***4***	89	51.8	
LAMC1	***4***	4	4	4	0	4	4	0	4	2	***4***	92	37.9	
FASLG	***4***	4	4	2	4	4	4	***4***	0	0	0	77	40.1	
HK2	***2***	4	4	4	3	0	3	0	4	4	***4***	94	46.5	2
ORC4L	***3***	3	3	4	3	3	3	0	3	***4***	3	88	33.2	
CCT7	***2***	4	2	2	1	4	4	4	2	2	***4***	95	46.2	2
FLVCR	***4***	4	4	4	4	0	4	1	0	4	0	77	39.0	1
ITGAV	***4***	4	0	4	4	***4***	4	4	0	0	0	91	36.4	
MYO1B	***4***	4	4	4	0	0	0	4	4	4	***4***	89	31.5	
RGS16	***4***	4	1	4	3	***4***	4	4	0	0	0	84	44.7	
PINK1	***4***	4	0	2	4	3	1	1	4	***4***	4	81	48.9	
EIF5B	***4***	4	4	4	3	0	0	4	3	1	4	75	38.7	1
GNB1	***3***	4	***4***	4	4	0	3	0	3	0	3	100	51.6	
GDAP2	***4***	4	3	4	0	0	0	3	4	4	***4***	94	35.0	
BIN1	***0***	3	3	3	0	3	3	3	0	3	***3***	69	60.8	3
WDR8	***4***	4	4	4	0	0	0	0	4	4	***4***	89	35.0	
AP4B1	***4***	4	4	0	4	0	0	0	3	4	***4***	90	39.0	
CYP2J2	***4***	4	1	4	3	3	0	2	0	2	0	76	43.7	1
GABRD	***0***	1	2	2	1	2	2	0	4	***4***	4	91	64.0	3
SULT1C1	***4***	4	4	4	4	0	0	0	0	2	0	83	45.9	1
RBBP4	***2***	2	2	2	2	2	2	0	2	2	***2***	99	38.8	2
DVL	***0***	0	4	2	2	2	2	0	2	2	***4***	95	69.6	3
SAC	***4***	0	0	4	4	0	0	0	4	***4***	3	73	45.0	
CCNT2	***0***	0	0	4	0	0	0	4	4	4	***4***	69	54.6	3
JAK1	***4***	0	0	4	0	0	3	***4***	4	0	4	93	52.4	
TBR1	***0***	3	0	2	0	1	1	2	3	3	***3***	99	53.4	3
WDR39	***1***	0	0	2	1	0	1	0	3	4	***4***	94	54.1	3
ARID4B	***1***	3	0	0	0	0	0	0	3	***3***	1	87	35.7	3
STAT1	***0***	3	0	0	0	0	0	0	0	***4***	4	92	39.9	4

^a^Boxes indicate index species used to design primers. Species abbreviations are: Hum, human; Mon, monkey; Hor, horse; Goa, goat; Ham, hamster; Mou, mouse.

^a^These numbers are the percent protein sequence identity between human and mouse.

^c^These numbers are the G + C content in the e-PCR amplified human product.

^d^Notes: 1, rabbit was the second index species for these primer pairs, except *FLVCR*, for which cat was used; 2, one or more processed pseudogenes apparent in the human genome; 3, contains a > 130 bp window in amplified human product with > 59% G + C; 4, strong primer-dimer seen in amplifications.

^e^Numbers 0–4 correspond to amplification rankings as exemplified in Fig. 1.

**Table 4:** PCR success vs. target GC content for zero IS-mismatch primer pairs

	dog			hamster		
		
GC range	no. tested	successful PCR	%	no. tested	successful PCR^a^	%
24–30	31	26	83.9	33	16	48.5
30–35	167	129	77.2	189	83	43.9
35–40	186	131	70.4	195	91	46.7
40–45	148	109	73.6	160	81	50.6
45–50	91	58	63.7	101	50	49.5
50–55	99	67	67.7	103	54	52.4
55–60	69	34	49.3	70	30	42.9
60–65	37	13	35.1	42	22	52.4
>65	10	2	20.0	12	4	33.3

^a^Successful hamster amplifications are based upon subjective rankings of 2, 3, and 4 (see Fig. 1 for the ranking system).
